# Paracrine Interactions between Mesenchymal Stem Cells Affect Substrate Driven Differentiation toward Tendon and Bone Phenotypes

**DOI:** 10.1371/journal.pone.0031504

**Published:** 2012-02-15

**Authors:** Ram I. Sharma, Jess G. Snedeker

**Affiliations:** 1 Department of Orthopedics, University of Zurich - Balgrist, Zurich, Switzerland; 2 Department of Mechanical Engineering, Swiss Federal Institute of Technology (ETH) Zurich, Zurich, Switzerland; University of Minho, Portugal

## Abstract

We investigated substrate dependent paracrine signaling between subpopulations of bone marrow stromal cells (BMSCs) that may affect the formation, or perhaps malformation, of the regenerating tendon to bone enthesis. Polyacrylamide substrates approximating the elastic modulus of tendon granulation tissue and the osteoid of healing bone (10–90 kPa) were functionalized with whole length fibronectin (Fn), type-I collagen (Col), or a mixed ligand solution (Fn/Col), and BMSCs were cultured in growth media alone or media supplemented with soluble Col or Fn. More rigid substrates with a narrow mechanical gradient (70–90 kPa) robustly induced osteogenic cell differentiation when functionalized with either Col or Fn. On broader mechanical gradient substrates (with a linear elastic modulus gradient from 10–90 kPa), cell differentiation was markedly osteogenic on subregions of Fn functionalized substrates above 20 kPa, but osteogenic activity was inhibited on all subregions of Col substrates. Osteogenic behavior was not observed when cells were cultured on Fn substrates if Col was present either in the media or on the substrate (Fn/Col). Tenogenic differentiation markers were observed only on Col substrates with moderate rigidity (∼30–50 kPa). Tenogenic differentiation was unaltered by soluble or substrate bound Fn. Co-culture of narrow gradient subsections revealed that any inclusion of tenogenic substrates (30–50 kPa, Col), caused otherwise osteogenic substrates to not develop markers of osteogenic differentiation, while increasing cell proliferation. These apparently paracrine effects could be mediated by bone morphogenetic protein-2 (BMP-2), as first confirmed by gene-level expression of BMP-2 and the transcription factor Smad8, and verified by BMP-2 media supplementation at levels similar to observed cell-secreted concentrations, which arrested osteogenic differentiation in 14 day cultures. Thus, cell instructive biomaterials with engineered mechanical and biochemical properties represent potentially powerful tools for directing BMSC differentiation to tendon and bone, however paracrine signals from tenogenic cells may delay osteogenesis at the healing enthesis.

## Introduction

The native tendon to bone junction is an exquisitely designed tissue interface comprising a cellular transition from the tendon itself, to a non-mineralized fibrocartilage region, to a mineralized fibrocartilage region, and ultimately to the bone [1], [2]. Post-traumatic healing of tendon to bone is generally poor, due in part to the competing objectives of a rapid recovery of joint function and the tissue complexity required for a mechanically robust interface [3]. BMSCs are highly relevant in the context of healing, being recruited to skeletal tissue damage as well as other major organs of the body including the heart, brain, liver, and skin [4]. Once recruited, BMSCs become actively involved in wound healing processes such as epithelialization, granulation tissue formation, and angiogenesis [5], [6], [7]. When a BMSC homes to an injury site, its behavior at the site is directed by a complex set of micro-environmental factors that include soluble and substrate-bound cues in the extracellular matrix, and intracellular signaling in the wound [8]. Homed BMSCs eventually participate in tissue repair in two manners: first by proliferation and eventual differentiation to appropriate numbers and phenotypes of cells required for healing, and second by mediating the behavior of cells involved in the repair process through paracrine signaling [9], [10]. BMSCs can secrete trophic factors that are highly stimulatory to tendon and bone extracellular matrix production and tissue remodeling, including growth factors such as transforming growth factor beta (TGF-β) and bone morphogenetic protein 2 (BMP-2) [11], [12], which can play a role in regulating differentiation and healing kinetics [13]. However, the interactions between BMSC paracrine signaling and extracellular matrix cues and how they affect progenitor cell differentiation at the healing tendon to bone interface remains to be elucidated.

We previously demonstrated that BMSCs could be differentially induced to commit toward bone and tendon cell lineages using engineered substrates of given ligand chemistry and mechanical compliance [14]. Here, biochemical and biomechanical cues were shown to regulate mitogen activated protein (MAP) kinase signaling, and directly affect gene level expression of transcription factors related to tenogenic and osteogenic differentiation [15], [16]. We utilized polyacrylamide hydrogels featuring a gradient of mechanical compliance spanning a range similar to granulation tissue. On these mechanical gradient substrates (MG substrates; spanning a range of moduli from 10–90 kPa) we focused on fibronectin as an extracellular matrix glycoprotein that plays a major role in cell dynamics like adhesion and migration during processes such as wound healing, development, and differentiation [17]. We also investigated type-I collagen, a key structural protein in both tendon and bone tissue [18] and the most abundant protein in skeletal connective tissues [19]. Although we were able to report for the first time induction of tenogenic differentiation using such substrates, we were unable to reach our original goal of creating localized regions of both tendon and bone-like cell lineages on the same gradient substrate; tenogenic cells were found only on the intermediate compliance region of collagen MG substrates (∼40 kPa), while osteogenic cells were increasingly localized on higher stiffness subregions of fibronectin MG substrates (above 40 kPa). It was particularly interesting to note that the stiffest regions of collagen MG substrates did not result in osteogenic cells as reported in previous studies using analogous systems with mechanically isotropic properties [20]. We conjectured that cell-cell contacts or paracrine signaling from various regions of the gradient stiffness substrate may have interfered with osteogenic differentiation in some cases, and thus prevented multiple cell lineages from forming on the mechanical gradient substrates.

In the present work we focused on the hypothesis that cell secreted cues can affect bone marrow stromal cell (BMSC) differentiation across remote sites of a MG substrate, and that these cues depend on both ligand chemistry and substrate stiffness. As before, we examined BMSC differentiation toward tenogenic and osteogenic lineages on gradient stiffness substrates functionalized with type-I collagen or fibronectin, but also explored mixed ligand functionalization, and the effects of stimulation by soluble fibronectin or collagen. To exclude the influence of cell-cell contacts across stiffness regions, we performed similar experiments using subsections of the broader mechanical gradient in the same culture well. We confirmed our hypothesis that cell-secreted (paracrine) cues from the tenogenic substrates could affect osteogenic differentiation within the system. We identified elevated signaling related to bone morphogenetic protein-2 (BMP-2) in tenogenic cultures that apparently interfered with (or delayed) the onset of osteogenic markers at the day 14 time point, on otherwise osteogenic substrates. Specifically, we found higher gene level expression of BMP-2 on MG30-50 (mechanical gradients with elastic moduli ranging from 30 to 50 kPa) collagen substrates, as well as on mixed and collagen substrates that were stimulated with soluble fibronectin. Direct measurements of BMP-2 secretion using immunosorbance techniques were substantially higher on collagen-presenting intermediate stiffness (i.e. tenogenic) substrates compared to fibronectin. Exogenous BMP-2 stimulation at levels similar to those observed in tenogenic cultures also interfered with differentiation on otherwise osteogenic substrates, while increasing cell proliferation. We thus conclude that BMP-2 secretion involved in BMSC differentiation can be modulated by strategic choice of cell substrate. BMP-2 signaling has been implicated in both osteogenic and tenogenic differentiation in vivo [21], and we have demonstrated that substrate regulated, paracrine BMP-2 signaling among local populations of BMSCs could have implications to healing at the tendon-bone interface where a tightly coordinated interaction of multiple cell phenotypes is required.

## Results

**Figure 1:**
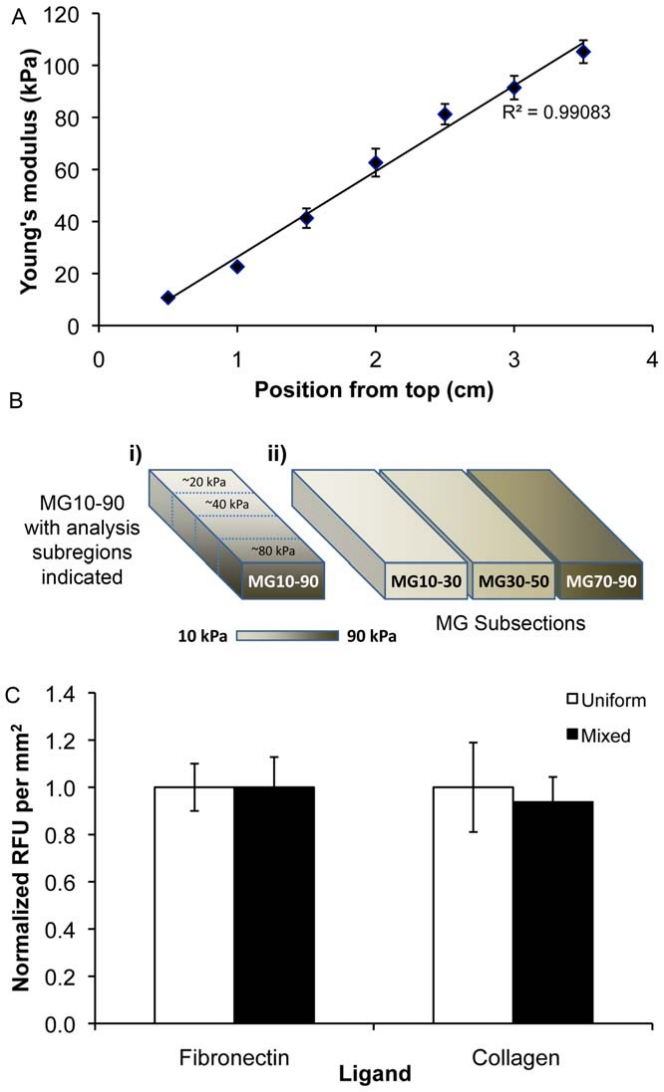
Characterization of cell culture interface. (A) The substrate compliance was measured in 0.5 cm increments using spherical indentation testing. Compliance was a linear function of longitudinal position. (B) Schematic representation of (i) MG10–90 broad gradient substrates with outlined subregions representing those analyzed after culture and (ii) narrow gradient subsection substrates. (C) Immunosorbance techniques were used to measure the bioactivity of substrates functionalized with fibronectin, collagen, or a mixed ligand solution of both. Activity was relatively similar between the uniform and mixed ligand conditions.

The mechanical compliance was determined to range from 10 to 150 kPa as a function of longitudinal position on the hydrogel substrate (Fig. 1A). As reported earlier, there was negligible change in mechanical properties after 14 days of cell culture compared to baseline readings [14]. As we used a mixed ligand solution (type-I collagen and fibronectin) and compared differentiation to collagen and fibronectin functionalized substrates, bioactivity of substrates was assessed using immunofluorescence to determine if differences between the uniform and mixed ligand existed. As shown in Fig. 1B, the normalized relative fluorescent units (RFUs) per unit area of sample did not vary between the uniform and mixed ligand conditions. These results indicate that using a mixed ligand solution did not compromise the ligand bioactivity compared to substrates functionalized with uniform ligand solutions.

**Figure 2:**
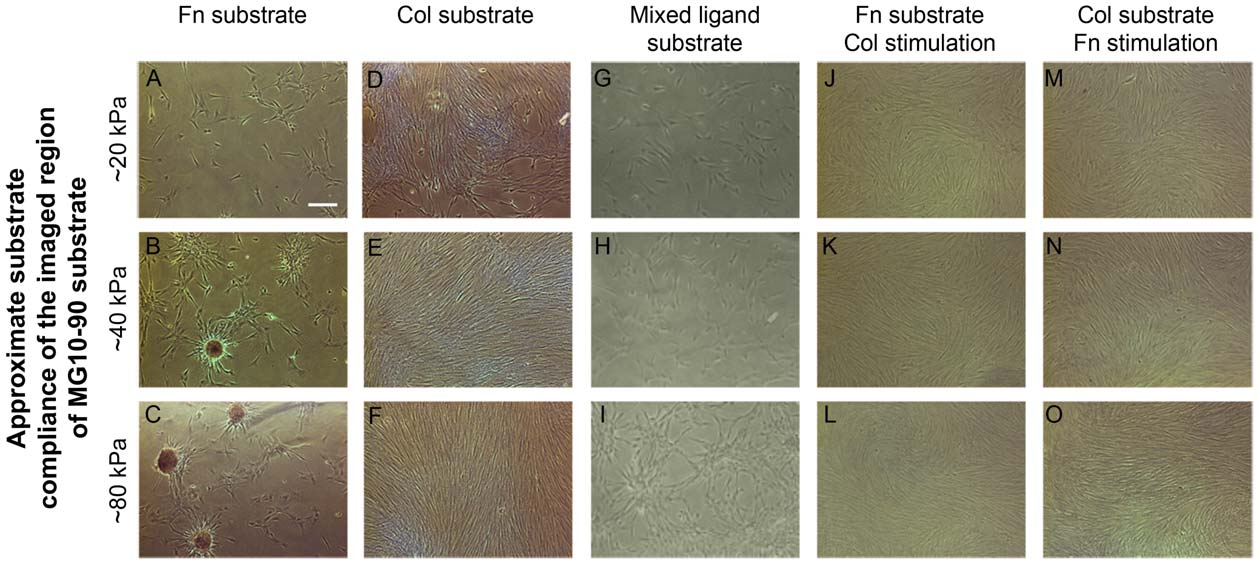
Differentiation from different culture conditions. Bone marrow stromal cells were cultured on MG10–90 substrates as described in the text for 14 days and processed for osteogenic behavior. (A–C) On fibronectin substrates osteogenic behavior was indicated by nodule formation on intermediate (∼40 kPa) and stiffer (∼80 kPa) subregions. (D–F) On collagen substrates osteogenic behavior was not seen. (G–I) When collagen was presented in the media, osteogenic behavior was not seen on fibronectin substrates. (J–L) Fibronectin media presentation did not appear to alter appearance of cells. (M–O) On mixed ligand substrates where the bulk ligand density was distributed using 20 µg/ml of total protein, osteogenic differentiation was not observed. Scale bar represents 100 µm.

We first probed BMSC differentiation on MG10-90 substrates (moduli ranging from 10 to 90 kPa) that were functionalized with fibronectin or type-I collagen at 10 µg/ml (Fig. 2). Cells were seeded in growth media, and after 14 days, samples were assessed for osteogenic differentiation with Alizarin Red staining of calcium present in the matrix. This assay indicated the presence of mineralized nodules on fibronectin functionalized MG10-90 substrates (Fig. 2A–C) that were not seen on collagen functionalized MG10-90 substrates (Fig. 2D–F). When a mixed ligand solution of type-I collagen and fibronectin (each at 10 µg/ml, resulting in a bulk protein solution concentration of 20 µg/ml) was used, nodules were not observed although a comparable amount of fibronectin was present on the MG10-90 substrate (Fig. 2G–I). Next, cells were seeded on fibronectin or collagen functionalized MG10-90 substrates, but cultured in growth media supplemented with type-I collagen for fibronectin functionalized MG10-90 substrates, or fibronectin for collagen functionalized MG10-90 substrates. Separate studies using media supplementation with FITC conjugated collagen indicated that while some soluble collagen precipitated onto the cells and passively bound to the extracellular matrix, most was actively assembled into the fibrillar matrix by the cells (data not shown). As with substrate bound type-I collagen, soluble collagen also apparently interfered with osteogenic differentiation (nodules were not seen on fibronectin MG10-90 substrates (Fig. 2J–L)). Consistent with our collagen functionalized MG10-90 substrates, we did not see nodules on collagen MG10-90 substrates when the media was supplemented with fibronectin (Fig. 2M–O).

**Figure 3:**
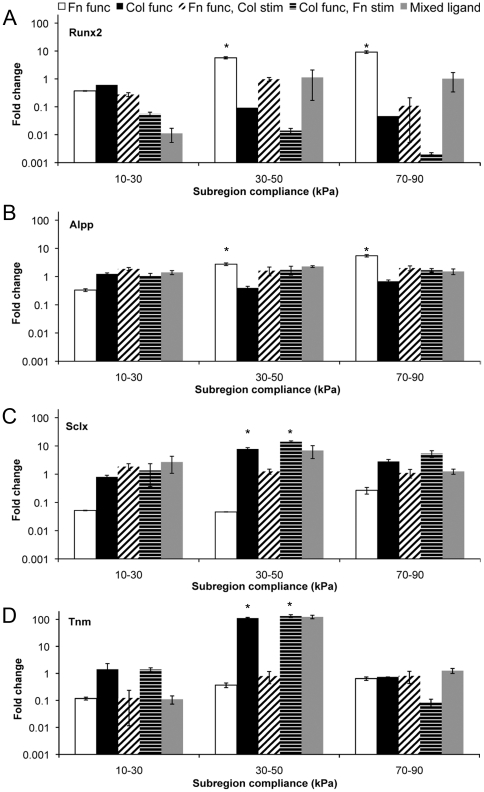
Markers of tenogenic and osteogenic differentiation from PCR. (A–B) Runx2 and alkaline phosphatase gene expression were similarly regulated, confirming the presence of osteogenic cells on fibronectin substrates in the intermediate and stiff subregions (∼40 and ∼80 kPa, respectively) of the intact MG10–90 substrate. (C–D) Scleraxis and tenomodulin expression confirmed tenogenic cells on the intermediate subregions of collagen functionalized substrates. * statistical difference (p<0.05) compared to fold change from the MG10–90 substrate at ∼20 kPa.

We confirmed osteogenic differentiation by using quantitative polymerase chain reaction (qPCR) to assess gene expression fold change of Runx2 and alkaline phosphatase (Alpp). Higher levels were seen on fibronectin MG10-90 substrates when cells were cultured with growth media (Fig. 3A–B). This was consistent with the Alizarin Red staining data. Fold changes comparable to controls (BMSCs cultured on TCPS), or in some cases a down regulation, were achieved on MG10-90 substrates for which collagen was present either on the substrate or in the media. We probed for tenogenic differentiation by assaying gene expression for scleraxis (Sclx), a transcription factor expressed by cells undergoing tenogenic differentiation, as well as tenomodulin (Tnm), a protein expressed by tenocytes (Fig. 3C–D). Expression of these two genes was altered on collagen presenting MG10-90 substrates but not fibronectin presenting MG10-90 substrates.

**Figure 4:**
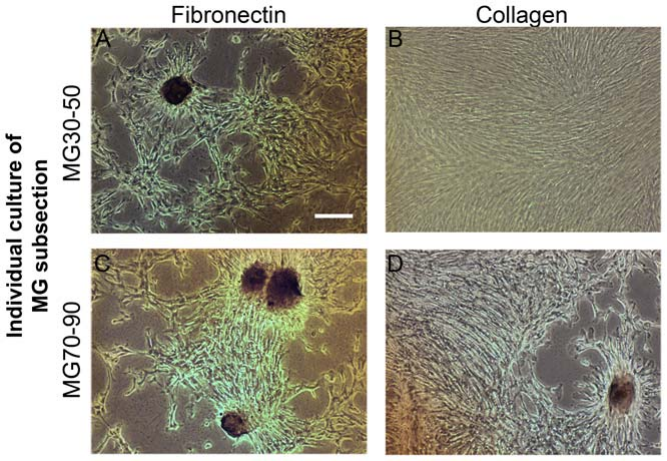
Differentiation on individual cultures of the gradient subsections. (A, C) Similar to MG10–90 substrates, osteogenic plaques were visualized on subsections of the fibronectin substrates. (B, D) Morphologically, on the MG30–50 substrate cells appeared similar to the corresponding region of the MG10–90 collagen substrate. However, osteogenic plaques were evident on the MG70–90 substrate. Scale bar represents 100 µm.

**Figure 5:**
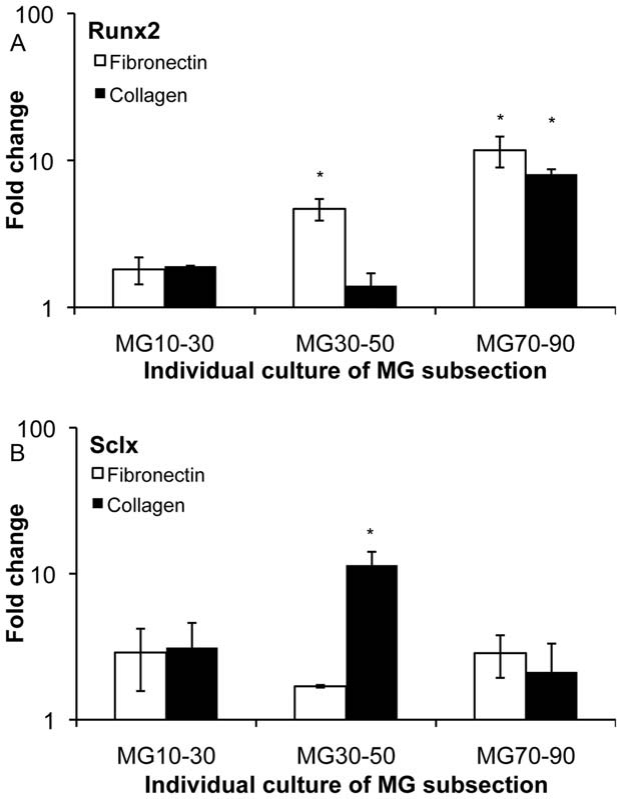
Molecular assessment similar to staining experiments. PCR assessed the expression of (A) Runx2 factor and (B) scleraxis, which was expressed with trends consistent with the staining data. * statistical difference (p<0.05) compared to MG10–30 culture condition.

In contrast to fibronectin functionalized MG10-90 substrates, the stiffest subregions of collagen functionalized MG10-90 substrates did not induce mineral plaques or gene level signaling related to osteogenic differentiation. We did confirm that collagen-functionalized subsections cut to isolate only the stiffest range of gradient (MG70-90) did induce osteogenic differentiation when cultured alone (Figs. 4,5). On fibronectin-substrates, mineral plaques and associated osteogenic gene signaling were observed on isolated mechanical gradients cultured independently using both MG30-50 and MG70-90. We further noted that gene expression on these fibronectin specific subsections was comparable to corresponding subregions of the MG10-90 substrate. In the case of subsectioned MG10-30 versus the corresponding subregion of the intact MG10-90 substrates, there was a slight discrepancy in gene expression: above one fold for MG10-30 subsections while analogous subregions of intact MG10–90 substrates indicated fold changes below one. We speculate that this may have been due to paracrine effects across subregions of the MG10–90 substrates that were absent in the MG10–30 subsection substrates.

**Figure 6:**
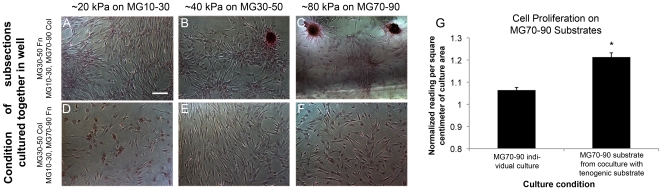
Culture of soft, intermediate, and stiff subsections of the gradient within the same well. (A–C) Osteogenic behavior was seen on the MG30–50 fibronectin substrate as well as the MG70–90 collagen substrate. (D–F) Morphologically the cells appeared similar to analogous subregions of MG10–90 collagen substrate studies, however osteogenic plaques were not visualized on MG70–90 fibronectin substrates. Scale bar represents 100 µm. (G) An increase in cell proliferation was observed on the osteogenic MG70–90 substrate (collagen functionalized) when co-cultured with a tenogenic substrate compared to the osteogenic substrate alone. * statistical difference (p<0.05) between the two conditions.

Based on these results, we investigated the hypothesis that tenogenic regions of the intact MG10–90 substrates (collagen functionalized substrates, regions corresponding to 30–50 kPa) were possibly interfering with osteogenic differentiation on stiffer subregions through paracrine signaling of growth factors and cytokines. To test this, we cultured subsections of the MG substrate in the same well spanning the same stiffness range to determine whether mineral plaques (osteogenic cells) could be obtained on stiff collagen subsections (MG70–90) when cultured in the same well as an intermediate compliance range (MG30–50) fibronectin functionalized subsections. Similarly, we cultured the stiff fibronectin subsection in the same well as the intermediate compliance collagen substrate subsections. In these studies, we found osteogenic plaques on the stiff collagen subsections (MG70–90) when cultured with the intermediate stiffness fibronectin subsection (MG30–50) (Fig. 6A–C). In contrast, osteogenesis was absent (lack of plaques) on the stiff fibronectin substrate sections when cultures of intermediate compliance collagen subsections (MG30–50) were present (Fig. 6D–F). The MG70–90 substrates also demonstrated increased cell proliferation behavior (Fig. 6G). These results supported the hypothesis that paracrine signaling was affecting substrate driven osteogenic differentiation within our system.

**Figure 7:**
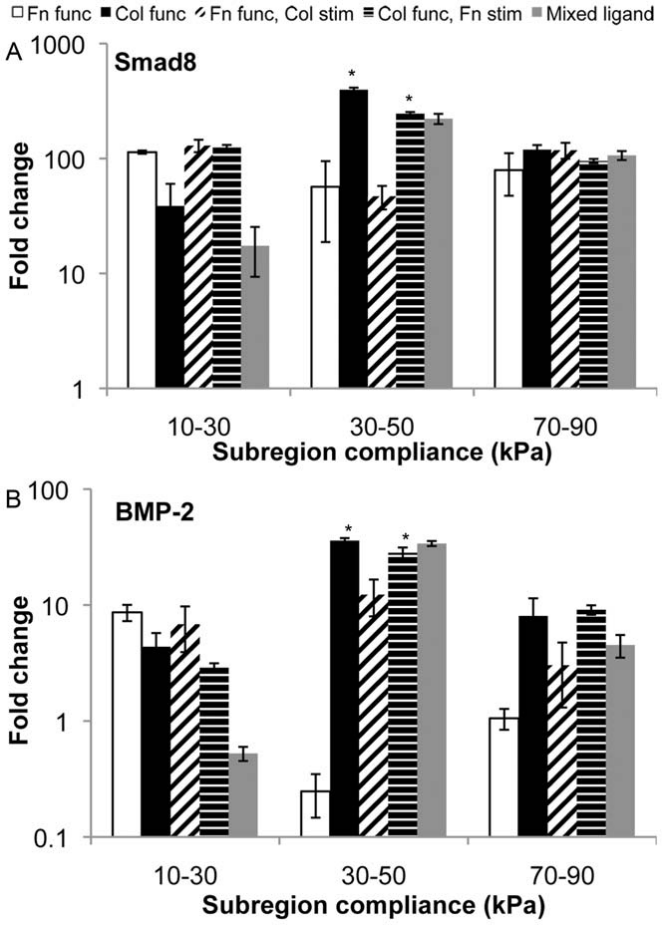
Smad8 and BMP-2 expression on MG10–90 substrates. (A–B) Smad8 and BMP-2 were measured for gene level expression, consistent with tenogenic gene expression (scleraxis and tenomodulin) trends on these substrates. * statistical difference (p<0.05) compared to fold change from the MG10–90 substrate at ∼20 kPa.

**Figure 8:**
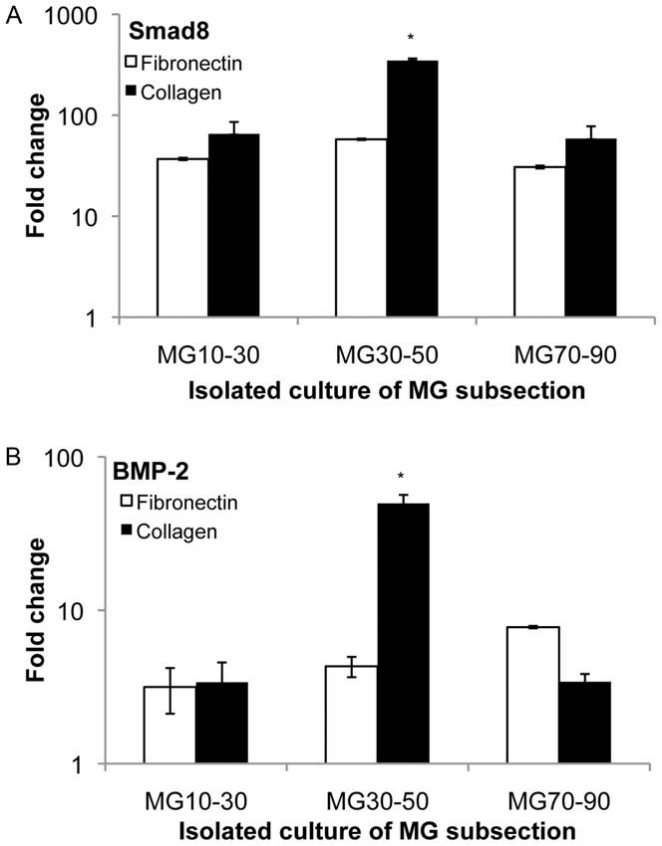
Substrate gene-level expression of Smad8 and BMP-2 from individual MG subsections. (A–B) Smad8 and BMP-2 were assessed from individual (narrow range) MG subsection cultures, also exhibiting trends similar to scleraxis from MG subsection studies described earlier, with maximum expression on the MG30–50 collagen substrate. * statistical difference (p<0.05) compared to MG10–30 culture condition.

**Figure 9:**
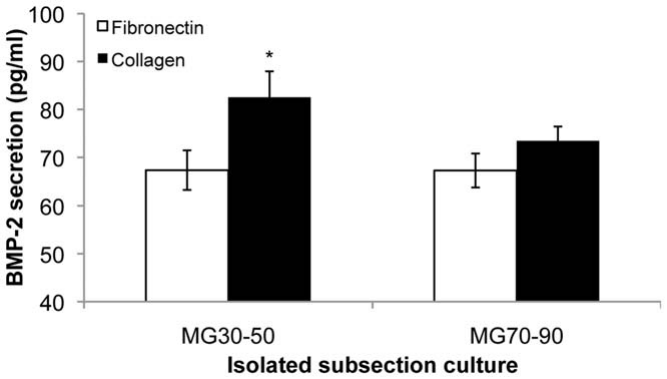
Assessing BMP-2 secretion. BMP-2 expression was assessed from individual cultures of MG subsections secreted in the media, which was expressed at a higher level on MG30–50 collagen substrates. * statistical difference (p<0.05) compared to 40 kPa fibronectin condition.

**Figure 10:**
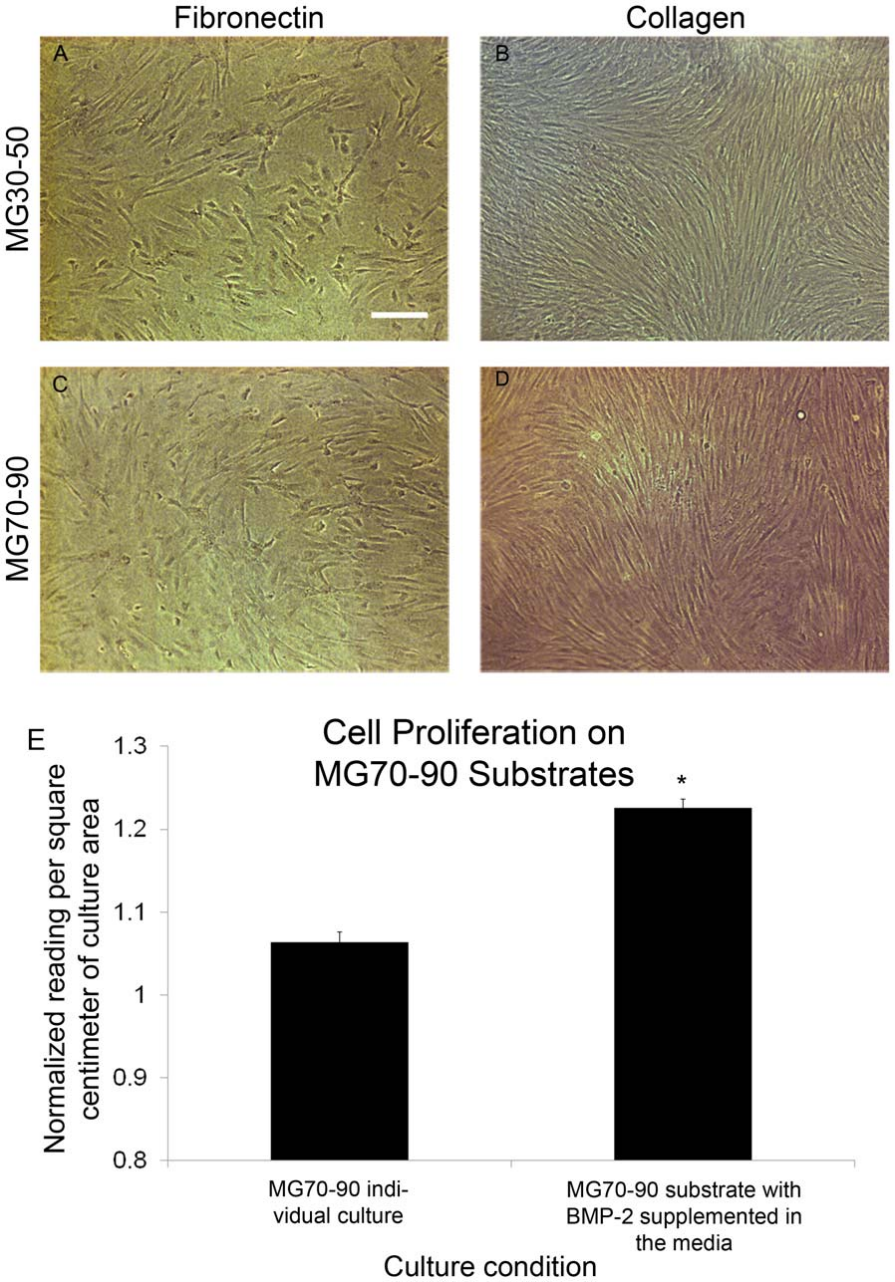
Arrest of osteogenic differentiation at day 14. (A–D) Individual culture of MG subsections supplemented with BMP-2 in the media no longer procured osteogenic plaques. Scale bar represents 100 µm. (E) The Alamar blue assay demonstrated an increase in cell proliferation on collagen functionalized osteogenic substrates (MG70–90) treated with BMP-2. * statistical difference (p<0.05) between two conditions.

Given its established primacy in tenogenic and osteogenic differentiation [22], [23], we then investigated signaling related to bone morphogenetic protein-2 (BMP-2). First we probed the expression at the gene level of both BMP-2 and Smad8, observing increased expression with qPCR on intermediate stiffness (MG30–50) collagen substrate sections compared to other conditions (Figs. 7,8). Secreted BMP-2 was confirmed by colorimetric assay, indicating increased presence of BMP-2 on substrates for which elevated gene expression of BMP-2 was observed (Fig. 9). We confirmed that BMP-2 supplementation at levels observed in isolated subsection culture was sufficient to arrest osteoblastic differentiation on substrate sections that were otherwise osteogenic at day 14 (Fig. 10 A–D), with an accompanying BMP-2 induced increase in cell proliferation (Fig. 10E).

## Discussion

Cells sense the biochemical and mechanical properties of their environment, and use these powerful cues to regulate their function [24]. In the case of multipotent progenitor cells, these cues can direct differentiation fate and kinetics [24]. At the molecular level, matrix dependent signaling occurs, including mitogen activated protein kinases (MAPKs), focal adhesion elements, Rho GTPases, and transcription factors, all of which can affect and modulate differentiation [16], [20], [25]. Previous reports have demonstrated that by culturing stem cells on substrates with moduli similar to those found in granulation tissue, differentiation fate can be controlled [14], [20]. Once differentiated or in the process of differentiating, cells begin to secrete growth factors and other proteins that can serve as soluble cues in either autocrine or paracrine fashions to affect downstream processes like extracellular matrix assembly [26], [27]. With a long-term view to apply these concepts in therapeutic strategies for improving tendon to bone healing [28], our earlier work on cell instructive culture substrates proved unable to simultaneously induce osteogenic and tenogenic differentiation of BMSCs on the same substrate [14]. We conjectured that cell-cell contacts or paracrine signaling from tenogenic cells may have interfered with osteogenic differentiation at remote sites of a mechanically inhomogeneous substrate. In the present work we investigated this hypothesis, and bring forth potential mechanisms for the observed interference in osteogenic differentiation.

Soluble and insoluble cues from the microenvironment regulate BMSC-mediated tissue modeling and remodeling in wound healing processes. The established roles of BMSCs in healing are twofold, whereby they first proliferate and differentiate to provide cells that can regenerate damaged tissue [9]. The second role regards paracrine signaling that coordinates local cellular response to injury [9], [10]. The secretion of growth factors, mitogens, and other soluble factors by BMSCs is thus central to processes such as matrix protein production, cell proliferation, and cell migration. How paracrine signaling among local cell populations in the system may affect progenitor cell differentiation by reducing sensitivity to mechanical and biochemical cues is an area that warrants investigation, and understanding potential intracellular interactions is essential to understanding both why natural tendon to bone healing processes are often inadequate, and how we may design therapeutic biomaterials to augment healing [29].

We tested and confirmed our hypothesis that broad mechanical gradient (MG) substrates (MG10–90) can elicit dramatic differences in bone marrow stromal cell (BMSC) differentiation compared to substrates with a narrower range (more uniform) of mechanical properties. These differences further depended on ligand chemistry and presentation. As in our previous work, we found that tenogenic BMSC differentiation would only robustly occur on mechanical gradient cell culture substrates within a narrow range of elastic moduli (30–50 kPa) [14]. We refined this insight by demonstrating that tenogenic differentiation further required the presence of substrate-bound type-I collagen, and that tenogenesis was not apparently inhibited by the presence of fibronectin in either substrate-bound or soluble forms. Conversely, osteoblastic differentiation on fibronectin substrates was inhibited by the presence of collagen in either substrate-bound or soluble forms. Interestingly, substrate-bound collagen could produce osteogenic cells, but only if the substrate was both sufficiently stiff (∼80 kPa) and there was no presence of tenogenic substrates within the culture well. Collectively these data suggest that tenogenic cells were interfering with osteogenic differentiation through paracrine signaling. Whether osteogenic differentiation is temporarily or indefinitely delayed (following the shift to a more proliferative state) will be the focus of future investigations into the signaling processes we discuss below.

Based on work by others demonstrating the sufficiency of induced Smad-8/BMP-2 signaling to provoke tenogenic BMSC differentiation [22], [23], we focused on BMP-2 signaling as a potentially key mediator of the observed behaviors. Perhaps the most novel finding of the current work is that substrate mediated BMP-2 signaling can affect BMSC proliferation and osteogenic differentiation, whether cell secreted or supplemented to the media. BMP-2 is part of the transforming growth factor-β (TGF-β) superfamily of proteins and binds to the BMP receptor. Upon its ligation with an appropriate ligand, various elements of the Smad family are expressed in the cytoplasm before translocating to the nucleus for the expression of appropriate transcription factors. BMP-2 is well characterized to enhance differentiation in two-dimensional systems, where stem cells are grown on plastic or glass substrates with other soluble cues including dexomethasone, L-ascorbic acid, and β-glycerophosphate, and its inhibition prevents osteogenic differentiation [30]. However, recent reports highlight the sensitivity of integrins and other cell surface receptors to substrate compliance. For instance, BMP receptor expression has been shown to increase on stiffer substrates, while softer substrates promote uptake of receptors via endocytosis and repress surface expression [31]. This change in surface expression could regulate cell sensitivity to BMP levels in the microenvironment, and this may underlie the observed shift to a more proliferative state rather than differentiating. BMPs have been implicated in increased cell proliferation [32], [33], [34], [35], with some of these studies suggesting that phosphorylated Smad activity after BMP receptor activation promotes proliferation. Additional investigation of substrate dependent BMP-2 signaling at various time points is required to elucidate these mechanisms.

In tenogenic differentiation, it is apparently necessary to activate scleraxis from Smad8; recent findings illustrate that BMP-2 is a critical factor in Smad8 expression, both from cell expressed BMP-2 as well as exogenous application in soluble form [22]. In our work, we activated scleraxis gene expression on collagen substrates possessing an intermediate bulk elastic modulus (30–50 kPa). As summarized above, substrate-bound collagen promoted tenogenic differentiation, even in the presence of substrate-bound or soluble fibronectin. Our previous studies showed higher degrees of BMSC attachment and spreading on collagen substrates relative to fibronectin [14]. Thus, in mixed ligand substrates (both collagen and fibronectin bound to the substrate), BMSCs may preferentially bind to type-I collagen. Whether this results in a down-regulation of fibronectin binding receptor (e.g. α_5_β_1_ integrin) number or engagement also represents grounds for future study. However, with soluble collagen in a substrate-bound fibronectin system, tenogenic differentiation did not occur. These findings align with investigations by Fan and co-workers that have shown that mesenchymal stem cells can respond differently to substrate presented ligand compared to soluble ligand, with differentially activated signaling pathways [36]. Our findings thus support that scleraxis and other transcription factors are critically regulated by ligand-integrin binding, with certain key effects of collagen-induced signaling not being triggered by soluble ligand presentation.

The culture of subsections within the same well performed in this study were designed to address paracrine signaling and rule out cell-cell contact involvement in the altered osteogenic differentiation patterns observed using subsections of the broader range MG substrates (MG10–90). Central to these studies were the tenogenic subsections (collagen functionalized MG30–50) and their influence on other substrate sections (or subregions of the MG10–90) by secretion of soluble cues. While we did include the MG10–30 substrate sections in the culture to more fully replicate the intact MG10–90 cultures, our reporting here focuses on the influence of the MG30–50 collagen functionalized substrate. As indicated by baseline levels of Runx2 and scleraxis expression in quantitative PCR analysis of the ∼20 kPa region of both intact and sectioned substrates, behavior of cells on the ∼20 kPa region of intact MG10–90 substrates differed somewhat from those on the isolated MG10–30 subsections – an effect we attribute to paracrine effects from other subregions of the intact substrates. However, we presume that these effects had little bearing on the interpretation of results from the culture of multiple subsections representing the overall mechanical gradient, since cell behaviors on stiffer substrates (40 kPa and above) were otherwise fully consistent between subregions of the intact MG10–90 substrates and analogous subsections with a narrower range of moduli.

While two dimensional in vitro cell and tissue culture systems remain an indispensable tool for shedding first light on the cell mechanics modulation of cell function [14], [20], [37], BMSCs in vivo are ensconced in a three-dimensional matrix composed of various proteins including fibronectin and collagen, and are further susceptible to cues from a large range of cell phenotypes resident in the tissue. In the present work, we assessed the role of paracrine signaling between cells stemming from a single phenotype within a highly-controlled two-dimensional system. While two-dimensional substrates can lack important spatial cues that affect cell signaling and behavior [38], analogous three-dimensional cell culture systems often lack quantitative control with regard to matrix mechanics and ligand presentation. Aside from three-dimensional cues, the present study also neglects transient mechanical loads that would act on the healing tendon-bone junction, and further exclude the influence that inflammatory or fibrocartilage cell phenotypes could have on system behavior. All of these simplifying limitations can be addressed in later (more complex) experimental designs, with the results of the present study laying a necessary foundation. In other words, we believe that the present study establishes important guiding principles for evolving biomaterial engineering approaches to study tenogenic and osteogenic differentiation of mesenchymal progenitor cells, particularly in terms of the interactions between relative matrix composition, matrix mechanics, and intracellular signaling. Accounting for these interactions in three dimensional systems with multiple cell types represents experimentally challenging but necessary future work.

In summary, our data show that BMSC differentiation can dramatically differ for cells cultured on substrates with broad regional differences in mechanical properties, compared to more narrow gradient substrate cultures. More specifically, tenogenic differentiation of bone mesenchymal stromal cells seems to interfere with osteogenic differentiation at a 14 day time point on otherwise osteogenic substrates, inducing a more proliferative state. This is apparently related to cell secreted cues, such as BMP-2, that may regulate intracellular coordination of stromal cell populations at the healing tendon to bone interface.

## Methods

### Substrate Characterization and Preparation

NuPage 4–12% electrophoresis gels (Invitrogen) were used as the substrate, with reproducible mechanical properties previously characterized and described by us [14]. The substrate has a nearly linear gradient of elastic modulus, with moduli ranging from 10 kPa to over 150 kPa. Regions with a range of moduli from 10 to 90 kPa (MG10–90) were sectioned and placed in a six well plate. For studies that examined individual subsections of the gradient, appropriate subsections were extracted, spanning a proportionally smaller region of the gradient. For studies that examined subsections within the same culture, subsections were sectioned and placed on a well with a small drop of Norland optical adhesive. The substrates were then exposed to UV light in a Stratalinker 2400 crosslinker (Stratagene) for 10 minutes. All substrates were then overlaid with sulfo-SANPAH (Pierce Biotech), a light sensitive crosslinker, at 0.5 mg/ml in 50 mM HEPES buffer (Sigma) and exposed to ultraviolet light in the crosslinker as previously described [37]. The crosslinker was removed and the substrates were overlaid with fresh crosslinker and exposed to ultraviolet light. The well plates were then covered and exposed to ultraviolet light for 15 minutes. At this point the substrates and well plates were sterilized and then brought back to the sterile field for further processing.

Fibronectin, collagen I (Sigma), or a mixed solution of collagen and fibronectin at 10 µg/ml each was overlaid, incubated overnight, washed, and used afterwards for cell culture experiments. Different regions of the MG substrates were previously found to have equivalent ligand distributions as determined from immunofluorescence [14], and the MG30–50 substrate was used in the present study for bioactivity immunosorbance assays for collagen and fibronectin compared to the mixed ligand solution. Functionalized substrates were blocked with 1% bovine serum albumin (Sigma) for 1 hour at 37°C, washed, and overlaid with either rabbit anti-human fibronectin diluted 1∶500 or mouse anti-collagen diluted 1∶1000 (Sigma) overnight at 4°C. Substrates were washed and overlaid with the appropriate FITC-conjugated donkey secondary antibody (Jackson Immunolabs) diluted 1∶200 for 1 hour at room temperature. Substrates were washed, sectioned, measured, and transferred to a 96 well plate to quantify relative fluorescence units (RFUs) on a Spectra Max GeminiXS (Molecular Devices). Isotherms were established by normalizing RFUs to area of samples. Experiments were performed in duplicates three times.

### Cell Culture

Bone marrow stromal cells (BMSCs) were a kind gift from Dr. Simon Hoerstrup's lab (University of Zurich) and had been first verified in that laboratory for multipotency in osteogenic, adipogenic, and chondrogenic induction assays [39]. In the cells used in these experiments, we verified an undifferentiated state by probing CD73, CD90, and CD105 as positive markers and CD34 as a negative marker using polymerase chain reaction (PCR) and normalizing expression to Gapdh. These multipotent stromal cells were cultured in growth media, based in alpha MEM (Invitrogen) and supplemented with 10% fetal calf serum and 1% penicillin/streptomycin (both Invitrogen). Media was exchanged every three days and maintained at 37°C with 5% CO_2_. Cells were used just before confluence. A cell suspension was created from near-confluent flasks by washing cells with PBS, overlaying with trypsin and neutralizing with growth media.

### Differentiation

A cell suspension was created as described above, and 25,000 cells/cm^2^ were seeded and cultured for 14 days at 37°C with media changes every three days. In some cases, media was supplemented with collagen or fibronectin at a final concentration of 10 µg/ml to examine the effect of soluble stimulation on fibronectin substrates or collagen substrates, respectively, as well as bone morphogenic protein-2 (BMP-2). At the 14 day time point, substrates were gently washed with PBS three times, and cells were fixed with 10% formaldehyde (Sigma) for 20 minutes at room temperature. The fixative was removed and substrates were carefully washed with distilled water three times. The substrates were then overlaid with 2% Alizarin Red (Sigma) (pH = 4.2) to stain for calcium present in the culture. The solution was incubated for 10 minutes at room temperature before being washed three times with distilled water. Images were acquired with a Nikon E600 upright microscope with a 10× objective. For each region of the MG10–90 substrate (20, 40, and 80 kPa) or individual subsections of the MG substrates and ligand/culture condition, at least five non-overlapping fields were imaged. Experiments were performed three times.

### Polymerase Chain Reaction

Two markers of osteogenic differentiation and two markers for tenogenic differentiation were evaluated at day 14 of stromal cell culture by implementing reverse transcriptase polyermase chain reaction (RT-PCR). Substrates were prepared as described above, BMSCs were cultured at 25,000 cells/cm^2^, and the total RNA was extracted using an RNeasy Kit (Qiagen) as per the manufacturer's protocols, and stored at −80°C until use. In the analysis of MG substrates (20–80 kPa), substrates were sectioned into three regions prior to isolating RNA. The cDNA was generated by using a cDNA Reverse Transcription Kit (Applied Biosystems). PCR was performed on resultant cDNA using SYBR green PCR Master Mix (SABiosciences) and upper and lower primers. Osteogenic markers probed included for alkaline phosphatase (Alpp), an enzyme expressed by osteoblasts [40], [41], runt-related transcription factor 2 (Runx2), a transcription factor for osteoblast differentiation [42], [43]. Scleraxis (Sclx), a transcription factor for tenoblasts [22], [44], [45], and tenomodulin (Tnm), a structural protein involved in collagen fibril maturation [46], [47], [48] were probed as markers of tenogenic differentiation. We also probed the gene level expression of BMP-2 and Smad8. Glyceraldehyde-3-phosphate dehydrogenase (Gapdh) was used as a housekeeping gene. A Roche Light Cycler (Roche), was utilized for fluorescent detection after each amplification cycle. The average threshold cycle number was used to calculate fold-changes in mRNA with control samples from cells cultured on tissue culture polystyrene from the control samples using the –ΔΔC_T_ [49].

### Alamar Blue Assay

The Alamar blue assay is designed to measure cell proliferation from different culture conditions by conversion of nonfluorescent resazurin to fluorescent resorufin in living cells [50]. BMSCs were seeded at 25,000 cells/cm^2^ on MG70–90 collagen subsections, MG70–90 collagen substrates supplemented with BMP-2 in the media, and coculture of MG30–50 and MG70–90 substrates. Cells were cultured for 4 days, at which point they were trypsinized to release cells, neutralized, centrifuged for 6 minutes at 900 rpm, and resuspended in 150 µl of media. The cell suspension was plated in a 96 well plate, along with two freshly seeded wells at a seeding density of 25,000 cells/cm^2^. Cells were given 24 hours to settle before treating with media supplemented with 10% Alamar blue reagent (Invitrogen) for an additional 24 hours. As a control, prior to Alamar blue treatment one well with untreated cells was fixed for 15 minutes with formaldehyde. Readings were taken on a Spectra Max GeminiXS fluorescent plate reader with 570 nm excitation and emission readings at 600 nm, standardized to substrate culture area and normalized to the emission reading for untreated cells on tissue culture polystyrene.

### BMP-2 Secretion

Bone morphogenic protein-2 (BMP-2) secretion was assessed using a colorimetric ELISA (R&D Systems). The ELISA uses well plates that are pre-coated with a monoclonal antibody specific for BMP-2. Briefly, a standard curve, ranging from 4 ng/ml to 0 ng/ml, was created from stock BMP-2 at 1 mg/ml and diluted with the provided dilution buffer. The standard curve and samples were overlaid in the pre-coated well plates with a provided assay diluent. The wells were incubated for two hours with agitation at room temperature, and washed four times with the provided wash buffer. Between each wash, the wells were shaken against paper towels and aspirated with a pipet to ensure complete removal of unreacted species. Next, a monoclonal antibody solution was prepared from the provided stock solution that binds to BMP-2 that is conjugated to an enzyme, overlaid on substrates, and incubated for two hours with agitation at room temperature. After washing substrates to remove any unbound antibody-enzyme reagent, a substrate solution was prepared from the provided kit and added to the wells. The color develops in proportion to the amount of BMP-2 bound in the initial step. The development is stopped with the stopping solution and read at 450 nm. Optical density readings were converted to concentrations from the best fit linear line to the standard curve.

### Statistics

All experiments were performed three times. Statistics were performed with single-factor analysis of variance (ANOVA), and a confidence level of 95% (p≤0.05) was considered necessary for statistical significance. All error bars represent the standard error around the mean.
